# Passive bilateral leg cycling with concomitant regional circulatory occlusion for testing mechanoreflex–metaboreflex interactions in humans

**DOI:** 10.1007/s10286-020-00717-x

**Published:** 2020-08-08

**Authors:** Adrian Lis, Wojciech Łopusiewicz, Massimo F. Piepoli, Beata Ponikowska, Bartłomiej Paleczny

**Affiliations:** 1 Department of Physiology, Wroclaw Medical University, ul. Chałubińskiego 10, 50-368 Wroclaw, Poland; 2 Department of Cardiology, Polichirurgico Hospital G Da Saliceto, Saliceto, Italy; 3 Institute of Life Sciences, Sant’Anna School of Advanced Studies, Pisa, Italy

**Keywords:** Exercise pressor reflex, Mechanoreceptors, Metaboreceptors, Passive cycling, Circulatory occlusion

## Abstract

**Purpose:**

The exercise pressor reflex (EPR) plays a fundamental role in physiological reactions to exercise in humans and in the pathophysiology of cardiovascular disorders. There is no “gold standard” method for EPR assessment; therefore, we propose a new protocol for testing interactions between the muscle mechanoreflex and metaboreflex (major components of EPR).

**Methods:**

Thirty-four healthy subjects (mean age [± standard deviation] 24 ± 4 years, 22 men) were enrolled in the study. During the study, the hemodynamic and ventilatory parameters of these subjects were continuously monitored using our proposed assessment method. This assessment method consists of an initial 5-min rest period (baseline) followed by 5 min of passive cycling (PC) on an automated cycle ergometer (mechanoreceptor stimulation), after which tourniquet cuffs located bilaterally on the upper thighs are inflated for 3 min to evoke venous and arterial regional circulatory occlusion (CO) during PC (metaboreceptor stimulation). Deflation of the tourniquet cuffs is followed by a second 5 min of PC and finally by a 5-min recovery time. The control test comprises a 5-min rest period, followed by 3 min of CO only and a final 5-min recovery.

**Results:**

Mean arterial pressure (MAP) and minute ventilation (MV) increased significantly during PC (MAP: from 90 ± 9.3 to 95 ± 9.7 mmHg; MV: from 11.5 ± 2.5 to 13.5 ± 2.9 L/min; both *p* < 0.05) and again when CO was applied (MAP: from 95 ± 9.7 to 101 ± 11.0 mmHg; MV: from 13.5 ± 2.9 to 14.8 ± 3.8 L/min; both *p* < 0.05). In the control test there was a slight increase in MAP during CO (from 92 ± 10.5 to 94 ± 10.0 mmHg; *p* < 0.05) and no changes in the ventilatory parameters.

**Conclusion:**

Bilateral leg passive cycling with concomitant circulatory occlusion is a new, simple and effective method for testing interactions between the mechanoreflex and metaboreflex in humans.

**Electronic supplementary material:**

The online version of this article (10.1007/s10286-020-00717-x) contains supplementary material, which is available to authorized users.

## Introduction

The exercise pressor reflex plays a fundamental role in physiological responses to exercise in humans [1–4]. It is triggered by the stimulation of muscle metaboreceptors, specifically by the metabolites produced when muscles contract, and by muscle mechanoreceptors, through the mechanical distortion of contracting muscles [1–3], and leads to sympathetic activation and vagal withdrawal that typically manifests as increases in cardiac output and blood pressure [1–3, 5]. These changes enable the human body to maintain perfusion pressure, thereby improving the blood supply to working muscles during exercise [6]. These effects, observed even in passive exercise, are reinforced during voluntary movements by the central mechanism, commonly known as “central command,” which is a complex feedforward mechanism originating in the brain [7]. The contribution of central command is a confounding factor and therefore should be excluded in experiments investigating the isolated role of muscle mechano- and metaboreflexes. Overactivity of the exercise pressor reflex has been associated with certain cardiovascular disorders, such as arterial hypertension and heart failure, and linked to exaggerated sympathetic outflow and hyperventilation, which are characteristics of these diseases [1, 2, 8–11].

Isolated responses from metaboreceptors can be measured with static exercise testing (e.g. handgrip test) and postexercise regional circulatory occlusion [8, 9], whereas the evaluation of muscle mechanoreflex sensitivity in humans remains a challenge. Several methods have been proposed, such as measuring passive movements [8, 14, 15], electrical stimulation [9, 16] and muscle stretching [17–20], but none of these are considered to be the “gold standard” due to inherent limitations. For example, the reproducibility of measurements of passive movements is a concern due to the measurements being performed by (different sets of) investigators or with custom-built equipment; electrostimulation evokes larger muscular metabolic perturbations than do voluntary movements [21–23]; and stretching muscles stimulate other types of mechanoreceptors than contracting muscles [1]. Even less is known about the interactions between the mechanoreflex and metaboreflex as very few studies have been performed [17–19, 24], all of which used the same muscle stretching protocol, and the results are highly discordant.

Here we propose a new method for testing the mechanoreflex–metaboreflex interactions in humans. The novelty of our method is that (1) it directly compares physiological responses to mechanoreceptor-only and mechanoreceptor–metaboreceptor activation; (2) it uses an exercise model resembling real-life activities (cycling); (3) it utilizes the “physiological” order of mechano- and metaboreflex stimulation (mechanoreceptors are stimulated first); and (4) it uses a commercially available, automated device for passive exercise (instead of custom-built devices or passive movements performed manually by a researcher). Although some of the aforementioned approaches considered separately are not novel (e.g. passive cycling [PC] has been used in an earlier study [14]), we are the first to address all of these factors simultaneously in a single experiment. The aim of this new strategy is to first induce isolated activation of the muscle mechanoreflex (using PC) and then to add metaboreflex stimulation (using circulatory occlusion [CO]) without stopping the PC so that both the mechano- and metaboreflexes are activated simultaneously. We hypothesized that PC alone would increase blood pressure and ventilation, that the subsequent addition of CO to PC would induce additional increases in these variables and that the values of these measures would return to the levels observed before PC and CO following cessation of these interventions.

## Methods

### Subjects

Thirty-four healthy volunteers participated in the study, of whom 22 were men. Mean age (± standard deviation [SD]), height, weight and body mass index (BMI) of the subjects was 24 ± 4 years, 178 ± 11 cm, 75 ± 15 kg and 23.7 ± 3.2 kg/m^2^, respectively. No previous histories of chronic disease were reported. The volunteers were asked to avoid intense exercise and drinking coffee for 24 h before the tests and eating food or smoking cigarettes for 2 h before the tests.

All subjects received detailed information about the study and gave written informed consent prior to participation. The protocol was approved by the local ethics committee. All procedures were performed according to the Declaration of Helsinki of 1964 and its later amendments.

### Experimental protocol and equipment

**Fig. 1:**
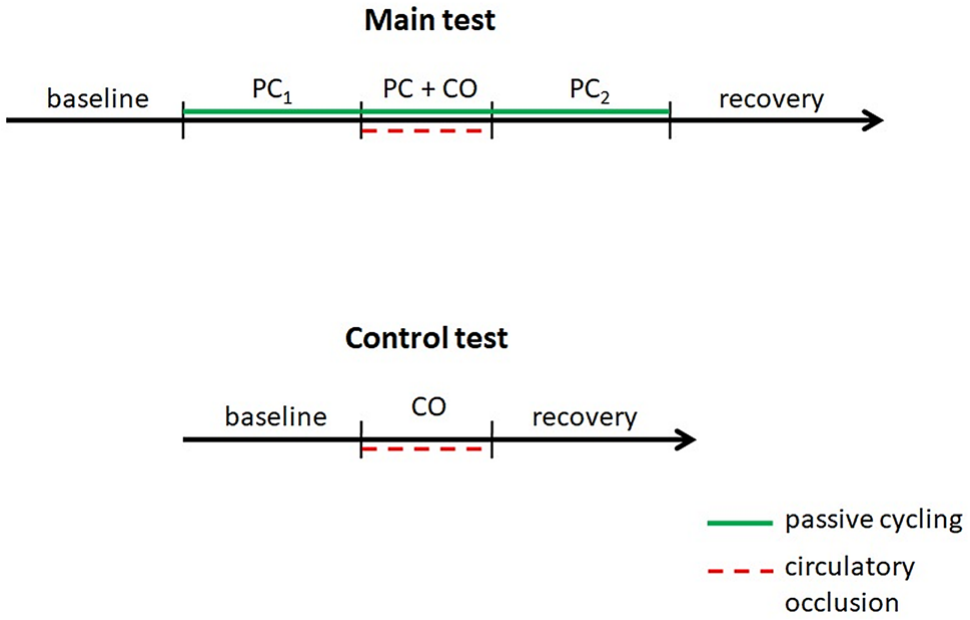
The protocol consists of two tests (main test and control test) performed in a random order. The main test consists of five consecutive phases: a 5-min resting period; 5 min of passive cycling (*PC*_*1*_); 3 min of circulatory occlusion during passive cycling (*PC + CO*); a second 5 min of PC after cessation of CO (*PC*_*2*_); and a 5-min recovery period. The control test comprises three consecutive phases: a 5-min resting period, followed by 3 min of CO and rounded off with a 5-min recovery period

The protocol consists of two tests: the main test and the control test (Fig. 1). During each test, hemodynamic and ventilatory parameters are continuously and noninvasively monitored and recorded. Heart rate (HR, in bpm) is calculated from lead II of the electrocardiogram (BioAmp; ADInstruments, Dunedin, New Zealand). Mean mean arterial pressure (MAP, mmHg), systolic blood pressure (SBP, mmHg) and diastolic blood pressure (DBP, mmHg) are recorded at a sampling frequency of 250 Hz using the Nexfin device (BMEYE B.V., Amsterdam, The Netherlands) and the volume-clamp method [25]. Stroke volume (SV, mL) and total peripheral resistance (TPR, dyn·s·cm^−5^) are calculated from the recorded pressures using a pulse contour method; higher TPR units reflect stronger peripheral vasoconstriction. Minute ventilation (MV, L/min) is calculated from instantaneous values of the breathing rate (breaths/min) and tidal volume (L), measured using a differential pressure transducer (FE141 Spirometer; ADInstruments) and a breathing circuit consisting of an oronasal face mask (Hans Rudolph, Inc., Shawnee, KS, USA) and a two-way nonrebreathing T-shape valve (Hans Rudolph, Inc.) connected to a flowhead (MLT3000 L; ADInstruments) positioned on the expiratory arm of the breathing circuit. All data are recorded with an acquisition system (PowerLab; ADInstruments) at a sampling frequency of 1 kHz. To ensure that the central command was not engaged, we used a commercially available automated cycle ergometer (Medbike, BTL, UK) that is certified for use in rehabilitation of individuals with neural disorders.

**Fig. 2:**
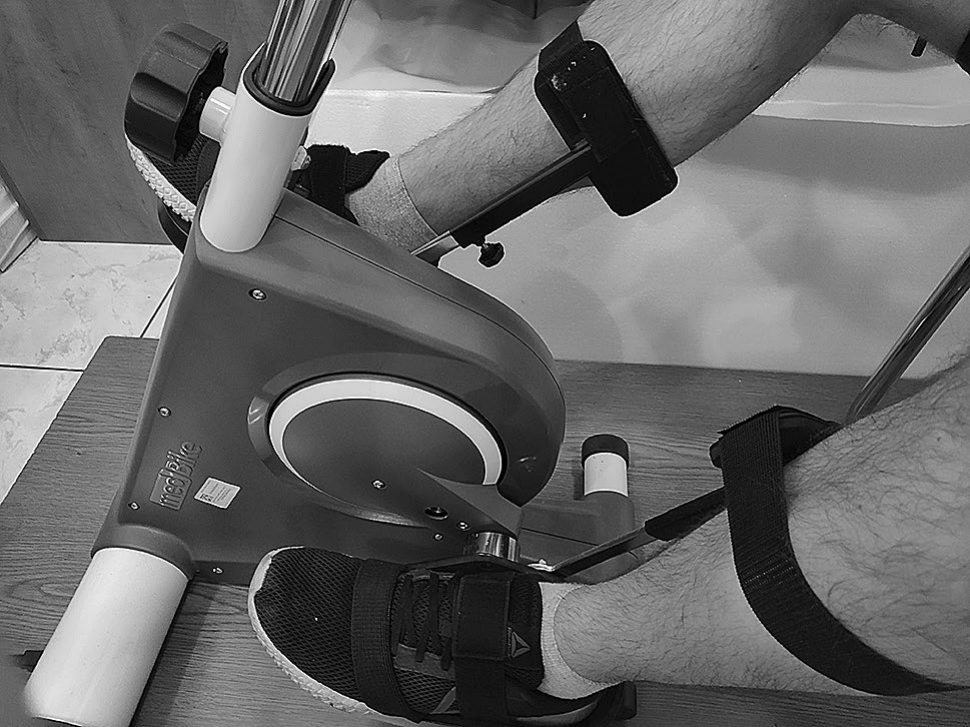
Rails stabilizing a subject’s feet

**Fig. 3:**
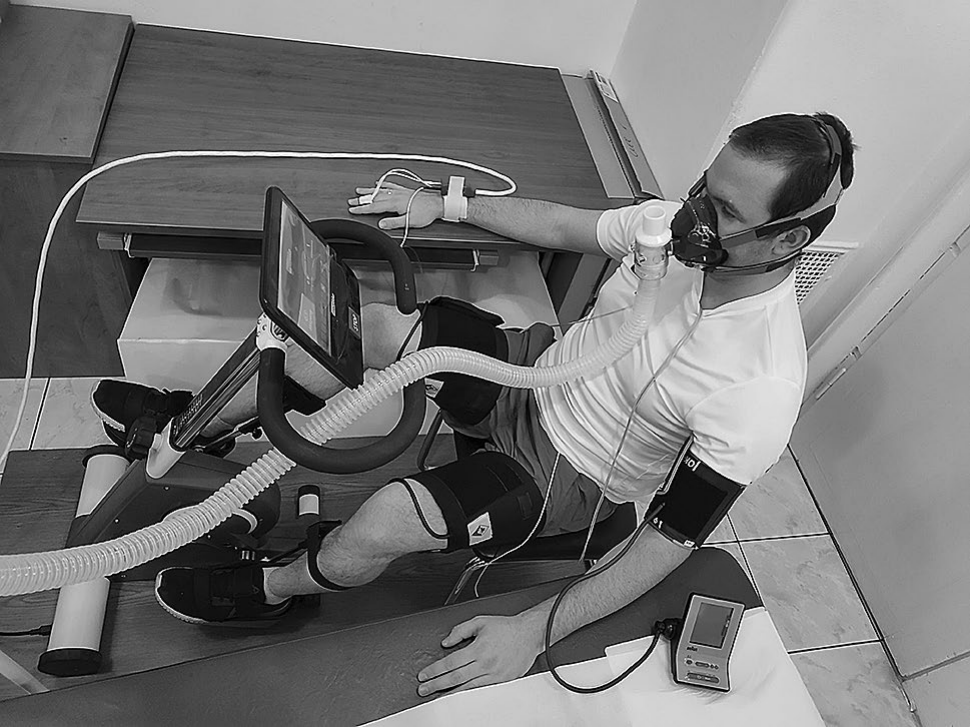
Laboratory set-up

The participants sit on the cycle ergometer with their knees flexed, their feet firmly attached to the pedals and their calves stabilized with rails connected to the pedals to prevent additional voluntary movements (Fig. 2). The distance between the seat and the ergometer is easily adjusted to ensure that there is a slight flexion in the knee when the foot is at its farthest point. The subjects are equipped with tourniquet cuffs attached bilaterally to their upper thighs during the entire protocol (Fig. 3). During the CO period of PC, the tourniquets are inflated (200 mmHg) to trap metabolites in the lower limbs. The pressure level selected is based on similar previously reported experiments [19, 26], taking into account gravitational factors [27] and allowing for the occlusion of venous and arterial circulation without evoking pain.

The main test comprises five consecutive phases: (1) a 5-min resting period; (2) 5 min of PC on the ergometer, with the pedaling rate set electronically at 60 rpm (mechanoreflex activation); (3) 3 min of bilateral upper thigh tourniquet cuff inflation to evoke venous and arterial regional CO during PC (activation of mechanoreflex and metaboreflex); (4) deflation of tourniquets and an additional 5 min of PC (mechanoreflex activation); and finally (5) a 5-min recovery period after cessation of PC. Each subject is instructed to relax and not to use any muscles during the course of study in order to minimize central command contribution. The participants are observed throughout the protocol to ensure they do not contract their muscles voluntarily.

The control test is performed to ensure that CO does not evoke hemodynamic or respiratory changes due to, for example, psychological factors or compression-induced activation of the muscle mechanoreflex. It comprises three consecutive phases: (1) a 5-min resting period; (2) 3 min of bilateral upper thigh tourniquet cuff inflation; and (3) a 5-min recovery period.

The tests are performed in a random order during the same visit in our laboratory. All participants rest for 15 min before the procedures. After the experiment, the subjects are asked to assess the pain in their legs during cuff occlusion (0–10 scale) and perceived exertion during PC using a Borg scale (6–20 points) [28].

### Statistical analysis

Data are presented as the mean ± SD. All percentage changes were calculated as the difference between the values from the two periods divided by the value from the first period. Hemodynamic and ventilatory parameters from each part of the two tests were analyzed with repeated-measures analysis of variance (ANOVA) and Duncan’s post hoc test. The two-way repeated-measures ANOVA was used to compare the differences between the main and control tests. *t* tests were used for group comparisons. A *p* value < 0.05 was considered to be significant.

## Results

**Fig. 4:**
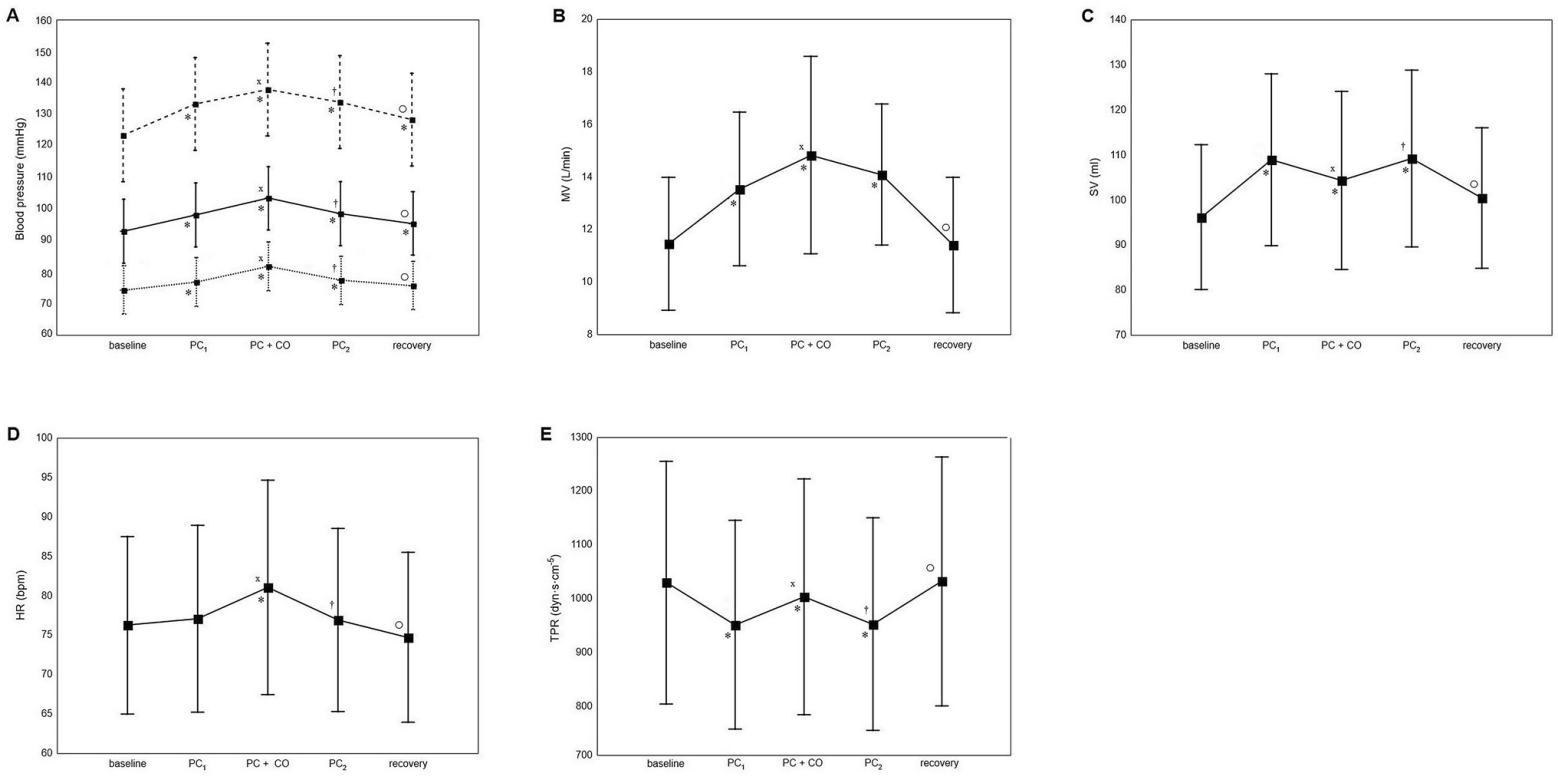
Plots showing the mean values and standard deviations of the mean arterial pressure (*MAP*; **a**, solid line), systolic blood pressure (*SBP*; **a**, dashed line), diastolic blood pressure (*DBP*; **a**, dotted line), minute ventilation (*MV*; **b**), stroke volume (*SV*; **c**), heart rate (*HR*; **d**) and total peripheral resistance (*TPR*; **e**) in consecutive test periods of the main test. See caption to Fig. 1 for description of the phases of the main test: baseline, PC_1_, PC + CO, PC_2_, recovery period. Changes in each parameter are statistically significant according to repeated measures analysis of variance when all test periods are considered together. *p < 0.05 between baseline and given period, ^X^*p* < 0.05 between PC_1_ and PC + CO, ^†^*p* < 0.05 between PC + CO and PC_2_, °*p* < 0.05 between PC_2_ and PC + CO

All hemodynamic and ventilatory parameters collected during the main and control tests are summarized in Tables 1 and 2, respectively. Plots for the main test are depicted in Fig. 4. During the main test, all parameters exhibited significant changes from baseline when all periods were considered together (all ANOVA *p* < 0.001). The changes in blood pressure and ventilation presented consistent patterns, including an initial increase in response to PC that increased further with CO. A different response was observed in SV, which increased during PC and then decreased slightly with CO. TPR followed the opposite pattern, decreasing with PC and slightly increasing during CO. HR was not affected by PC, but increased during CO. After PC and CO were stopped, the parameters tended to return to the values observed before the respective interventions (see also Electronic Supplementary Material). A slight increase in MAP, DBP, TPR, but no significant changes in the other parameters measured were observed during the control test. The gain in MAP and DBP in the control test was smaller than that in the main test (both *p* < 0.01), while the increase in the TPR was similar between the two tests (*p* = 0.19). There was no difference in self-reported leg pain between the main and control tests (3.5 ± 1.9 vs. 3.6 ± 1.8 on a scale of 10; *p* = 0.72). The rating of perceived exertion in the main test during PC was 6.3 ± 1.1 (range 6–20 points).

**Table 1:** Hemodynamic and ventilatory parameters recorded in the five consecutive phases of the main test

Hemodynamic and ventilatory parameters^a^	Period of main test^b^					*p* value*			
	Baseline (5-min resting period)	PC_1_	PC + CO	PC_2_	Recovery	Baseline vs. PC_1_	PC_1_ vs. PC + CO	PC + CO vs. PC_2_	PC_2_ vs. recovery
MAP (mmHg)	90 ± 9.3	95 ± 9.7	101 ± 11.0	96 ± 9.8	93 ± 9.8	< 0.001	< 0.001	< 0.001	< 0.001
SBP (mmHg)	120 ± 14.1	130 ± 14.4	135 ± 15.7	131 ± 14.6	125 ± 13.3	< 0.001	< 0.001	0.002	< 0.001
DBP (mmHg)	72 ± 7.0	75 ± 7.4	80 ± 8.5	75 ± 7.4	74 ± 7.5	< 0.001	< 0.001	< 0.001	0.02
TPR (dyn·s·cm^−5^)	1030 ± 225	952 ± 194	1003 ± 219	953 ± 197	1032 ± 231	< 0.001	< 0.001	< 0.001	< 0.001
SV (mL)	96 ± 16.1	109 ± 19.1	104 ± 19.7	109 ± 19.6	100 ± 15.6	< 0.001	< 0.001	< 0.001	< 0.001
HR (bpm)	76 ± 11.2	77 ± 11.8	81 ± 13.6	77 ± 11.6	75 ± 10.8	0.43	< 0.001	< 0.001	< 0.001
TV (L)	0.80 ± 0.3	0.84 ± 0.4	0.93 ± 0.5	0.87 ± 0.3	0.80 ± 0.3	0.49	0.07	0.17	0.22
BR (bpm)	15.3 ± 3.6	17.7 ± 4.0	17.3 ± 3.8	17.5 ± 3.9	15.4 ± 3.5	< 0.001	0.43	0.60	< 0.001
MV (L/min)	11.5 ± 2.5	13.5 ± 2.9	14.8 ± 3.8	14.1 ± 2.7	11.4 ± 2.6	0.02	< 0.001	0.16	< 0.001

Hemodynamic and ventilatory parameters in table are presented as the mean value ± standard deviation (SD) for each consecutive phase of the mean test

*Changes in each parameter are statistically significant according to repeated measures analysis of variance (ANOVA), when all phases are considered together. Note: for TV only, baseline vs. PC + CO and PC + CO vs. recovery are statistically significant according to Duncan’s post hoc test (*p* = 0.017 and *p* = 0.014, respectively)

^a^*MAP* Mean arterial pressure,* SBP* systolic blood pressure,* DBP* diastolic blood pressure,* TRP* total peripheral resistance,* SV* stroke volume,* HR* heart rate,* TV* tidal volume,* BR* breathing rate,* MV* minute ventilation

^b^PC_1_ is the first passive cycling (PC) period; it follows baseline (the initial 5-min resting period) and is followed by passive cycling with circulatory occlusion (PC + CO). Once CO ceases, PC continues (PC_2_). The last period is the recovery period. For full description, see section Experimental protocol and equipment

**Table 2:** Hemodynamic and ventilatory parameters recorded in the three consecutive phases of the control test

Hemodynamic and ventilatory parameters	Period of control test^a^			*p* value*	
	Baseline	CO	Recovery	Baseline vs. CO	CO vs Recovery
MAP, mmHg	92 ± 10.5	94 ± 10.0	92 ± 8.9	0.001	0.001
SBP, mmHg	123 ± 14.7	125 ± 13.7	123 ± 11.8	0.06	0.65
DBP, mmHg	73 ± 8.1	75 ± 7.8	73 ± 6.9	< 0.001	0.52
TPR, dyn·s·cm^−5^	1024 ± 216	1061 ± 218	1035 ± 220	< 0.001	0.26
SV, mL	100 ± 14.9	99 ± 15.1	98 ± 14.3	0.45	0.70
HR, bpm	76 ± 10.1	76 ± 11.7	75 ± 11.2	0.79	0.63
TV, L	0.78 ± 0.3	0.86 ± 0.4	0.82 ± 0.3	0.10	0.46
BR, bpm	15.4 ± 3.6	15.6 ± 3.5	15.6 ± 3.6	0.67	0.71
MV, L/min	11.2 ± 2.3	12.4 ± 3.6	11.9 ± 2.8	0.06	0.33

Hemodynamic and ventilatory parameters in table are presented as the mean value ±  SD for each consecutive phase of the control test

*Changes in the SBP, SV, HR, TV, BR and MV are not significant statistically according to repeated measures ANOVA when all periods are considered together

^a^Baseline period (5-min resting period) is followed by CO, which is turn is followed by the recovery period. For full description, see section Experimental protocol and equipment

## Discussion

The aim of this study was to develop a new, safe, simple and automated method for testing mechanoreflex–metaboreflex interactions in humans. We hypothesized that (1) PC alone affects hemodynamic and ventilatory parameters and, in particular, increases blood pressure, SV and MV (mechanoreflex activation); (2) adding CO to PC leads to additional changes, such as additional increases in blood pressure and MV (metaboreflex activation); and (3) once PC and CO cease, these parameters tend to return to the values observed before the aforementioned interventions. The results of this study support our hypotheses. Therefore, for the first time, we present a novel protocol that is effective in stimulating mechano- and metaboreceptors simultaneously and physiologically in a single experiment, using commercially available equipment.

The first step of this study was to choose the optimal technique to stimulate the mechanoreflex. Various methods have been used in earlier studies, such as involuntary muscle contractions with the use of electrostimulation [9, 16]; muscle stretching performed by investigators [17, 19] or by applying custom-built devices [18, 20]; passive movements on tandem bicycle [15] or on custom-built equipment and cycle ergometers [8, 14]. All these methods were designed to reduce the possibility of metabolite accumulation, which can stimulate metaboreceptors, and to eliminate voluntary movements that engage central command. However, electrostimulation and muscle stretching appear to be less reliable than passive movement. The major concern with using electrostimulation is the potentially confounding effect of concomitant activation of muscle metaboreceptors, as it has been shown that electrostimulation induces metabolite production [21–23]. This effect may explain the highly discrepant observations across studies targeting small muscles (slight increase in MAP, no effect on HR) [9] and large muscles (large increase in MAP and HR) [16]. The effects of muscle stretching are also difficult to interpret, given that stretching and contracting muscles stimulate different types of mechanoreceptors [1]. Moreover, stretching does not mimic the physiological model of dynamic exercise.

The results of the few studies that have used stretching to stimulate mechanoreceptors are highly variable. Depending on the type of a muscle, stretching induced an increase in MAP without affecting HR (in agreement with our findings) [20] or a decrease in MAP with an increase in HR [17]. The latter result is inconsistent with those of other studies reporting that mechanoreceptor stimulation induces an increase in MAP [9, 14–16, 20, 29]. Taking these concerns into account, we decided to choose PC to induce the mechanoreflex. This technique was first used by Nobrega et al. [29] and caused elevations in MAP and SV. Similar results were observed in all subsequent experiments involving PC, regardless of whether a tandem bicycle [15] or ergometer was adapted [14]. The technique has also been shown to be applicable for patients with heart failure, in whom it evoked MV increases [26].

Our results confirm that PC stimulates mechanoreceptors, which—most notably—increased SBP (8%; from 120 ± 14 mmHg at baseline to 130 ± 14 mmHg at PC), SV (13%; from 96 ± 16 mL at baseline to 109 ± 19 mL at PC) and MV (17%; from 11 ± 2 L/min at baseline to 13 ± 3 L/min at PC). All parameters decreased after PC was stopped, which suggests that hemodynamic and ventilatory changes persist as long as the mechanoreceptors are stimulated. We used a fully automated and commercially available, adjustable cycle ergometer, originally designed for rehabilitation for paresis and paralysis in lower extremities; it may become the standard device for testing the mechanoreflex.

The second step of this study was to create a valuable method for testing mechanoreflex–metaboreflex interactions in humans. Virtually all protocols used in previous studies to test interactions between mechanoreceptors and metaboreceptors involved muscle stretching [17–19, 24] in the following order of tasks: (1) exercise was performed, (2) post-exercise CO was applied and (3) the muscle was stretched. Aside from the issues with muscle stretching mentioned before, this approach also does not engage both reflexes in a physiological and simultaneous manner. Rather, it creates an interim metabolic background that overlaps with stretching. Our protocol implements a completely different paradigm. First, we used PC, which is effective in stimulating isolated mechanoreceptors and evokes consistent cardiovascular and ventilatory effects; second, we introduced a different order of interventions, wherein CO is applied on the limbs that are being moved passively. This method imitates the physiological stimulation of particular receptors during physical activity. In general, the first response comes from mechanoreceptors, and then, when metabolites accumulate, the metaboreflex starts to induce hemodynamic changes [1, 7].

Previous studies on mechanoreflex–metaboreflex interactions yielded inconsistent results, even when the same methods and muscles were used. Stretching of the calf muscle during CO in one study resulted in an increase in SV and HR without any change in MAP [18], whereas in another study it induced only a small increase in DBP [19]. In yet another study, isolated wrist stretching did not result in any changes in hemodynamic parameters, with only a small increase in MAP occurring when CO was added to stretching [24]. Using our protocol, we observed that almost all of the tested parameters showed regular, characteristic patterns in response to PC and CO. Additional and significant increases in blood pressure and MV were induced by CO during PC, indicating that the stimulation of metaboreceptors resulted in an additional response. These observations suggest our new protocol is a reliable tool for testing mechanoreflex–metaboreflex interactions in humans, with greater validity than protocols based on muscle stretching.

Interestingly, we observed a pattern of changes in HR that suggests hyperadditive interactions between the metabo- and mechanoreflex. Specifically, HR was not affected by PC or CO separately, but it increased when CO was added to PC. A lack of HR response to PC has been shown previously [14, 29]. Similarly, the isolated stimulation of metaboreceptors does not produce essential increases in HR [7]. The potential influence of the metaboreflex on HR is masked, even during CO, by parasympathetic reactivation, mainly due to a loss of central command [30–32]. Barbosa et al. [33] showed that blockade of the exercise pressor reflex attenuates the HR response to active cycling, which indicates that the increase in HR is also not caused solely by central command. Considering these facts and the results of our study, we can conclude that the aforementioned increase in HR is probably a unique example of cooperation between the mechanoreflex and metaboreflex. Thus, to evoke a substantial increase in HR, both mechanoreceptors and metaboreceptors must be stimulated. The aggregate feedback from both types of receptors is essential to provide a sufficient stimulus that meets the threshold for a change in HR.

Finally, the exaggerated exercise pressor reflex in heart failure and hypertension is presumed to be a relevant component of autonomic dysfunction, which is characteristic of these disorders [1, 2, 8–11]. Regarding the global character of these conditions, it is extremely important to comprehensively understand the precise mechanisms of deterioration in the metabo- and mechanoreflex. This knowledge may enable development of targeted therapies for cardiovascular diseases, based on selective blockade of the components of the exercise pressor reflex [34] and specific exercise training [1]. Thus, introducing a new reliable method for testing the metabo- and mechanoreflex is essential for clinical purposes as well.

## Limitations

It is difficult to eliminate voluntary muscle contractions when an individual’s legs are being moved by an ergometer. To prevent this disturbance and stabilize subjects’ calves, we use rails connected to the ergometer pedals (Fig. 2). The rails are standard parts of the ergometer. The subjects are also asked to relax, not to perform any movements and not to think about exercise. We did not notice any voluntary movements in the subjects throughout the protocol, and the mean physical effort rating on the Borg scale was very low. However, electromyography signals from the leg muscles were not recorded and thus central command contribution cannot be entirely excluded. We attributed the slight increase in MAP, DBP, and TPR in the control test to the modest accumulation of metabolites at rest and stimulation of metaboreceptors, although other factors cannot be excluded. It is unlikely that these changes were caused by pain, since we found no difference between the severity of pain in the limb caused by CO between the main and control tests.

## Conclusions

Our protocol has, for the first time, introduced the application of CO during PC, which allows us to mimic a physiological model of dynamic exercise. With the use of this scheme, we confirmed our hypothesis that PC evokes increases in blood pressure, SV and MV (mechanoreflex activation); adding CO to PC induces additional increases in blood pressure and MV (metaboreflex activation); and these parameters return to the values observed before PC and CO after these interventions are stopped. Our model has several advantages since it involves a safe, simple and automated method of stimulating mechanoreceptors in a physiological way and, additionally, stimulating metaboreceptors without terminating mechanoreceptor activation. We believe that this method will provide new opportunities to study interactions between these two components of the exercise pressor reflex, which is an intriguing area of scientific research but remains to be fully elucidated.

## Electronic supplementary material

Below is the link to the electronic supplementary material.

Supplementary file1 (DOCX 795 kb)
