# Supplementary material for: Passive bilateral leg cycling with concomitant regional circulatory occlusion for testing mechanoreflex–metaboreflex interactions in humans
Source: Clin Auton Res. 2020 Aug 8;30(6):549–56. doi: 10.1007/s10286-020-00717-x (PMC7704518; doi:10.1007/s10286-020-00717-x)
Supplement: Supplementary file 1 — Supplementary file1 (DOCX 795 kb) [file 10286_2020_717_MOESM1_ESM.docx]

**Supplementary material**

The following figures show one-minute epochs for each tested variable in a single representative individual. Each figure consists of graphs for the main test and control test. The main test comprised a 5-minute resting period; 5 minutes of passive cycling on the ergometer (PC, green line); 3 minutes of bilateral upper thigh tourniquet cuff inflation to evoke venous and arterial regional circulatory occlusion (CO, red line); deflation of the tourniquets and 5 more minutes of PC; a 5-minute recovery period after PC was stopped. The control test comprised a 5-minute resting period; 3 minutes of CO (red line); and a 5-minute recovery period.

The mean (MAP), systolic (SBP), and diastolic (DBP) blood pressures are depicted in the Fig. 1. All these parameters followed consistent pattern of changes. In the main test, at the early stage of PC, blood pressures increased markedly and then started to decrease slightly. They increased significantly again, shortly after CO was applied. This increase persisted approximately one minute after CO was stopped. Then the MAP, SBP and DBP decreased and remain relatively stable during PC. Blood pressures decreased markedly again when PC was stopped. There were slight increases in the MAP and DBP during CO in the control test. They returned to the baseline values after CO was stopped. There were no significant changes in SBP in the control test.

Different responses were observed in the total peripheral resistance (TPR, Fig. 2). In the main test, PC resulted in a sharp decrease in the TPR. During 5 minutes of PC, the TPR tended to increase, but the relevant gain was seen after approximately one minute of CO. The TPR decreased again after CO was stopped and sharply increased after PC was stopped. In the control test, the TPR increased after one minute of CO and decreased after CO was stopped.

The stroke volume (SV, Fig. 3) followed the exact opposite pattern in the main test. A substantial increase was seen in response to PC and a minor decrease was seen when CO was added. These responses persisted as long as the aforementioned interventions did. In the control test, CO did not change the SV value.

In the main test, we observed a transient peak in the heart rate (HR, Fig. 4) shortly after PC was applied, although it decreased quickly, so that there were no significant difference between the resting and PC periods. The HR increased markedly during CO period and decreased after CO was stopped, and then decreased again after PC was stopped. In the control test, there were no relevant changes in the HR between consecutive periods, although we observed another transient peak in the HR after CO was stopped.

The tidal volume (TV) is depicted in the Fig. 5. In the main test, the TV tended to increase in the response to PC and CO, although the values varied between consecutive minutes of the test. In the control test, we found no relevant changes in response to CO.

The breathing rate (BR, Fig. 6) and minute ventilation (MV, Fig. 7) followed a very similar pattern. In the main test, both parameters increased sharply during PC and then increased again after CO was applied. After PC and CO were stopped, the parameters returned to the values observed before the respective interventions. The BR and MV were not affected by CO in the control test.

**Figure 1 Time course of blood pressure**


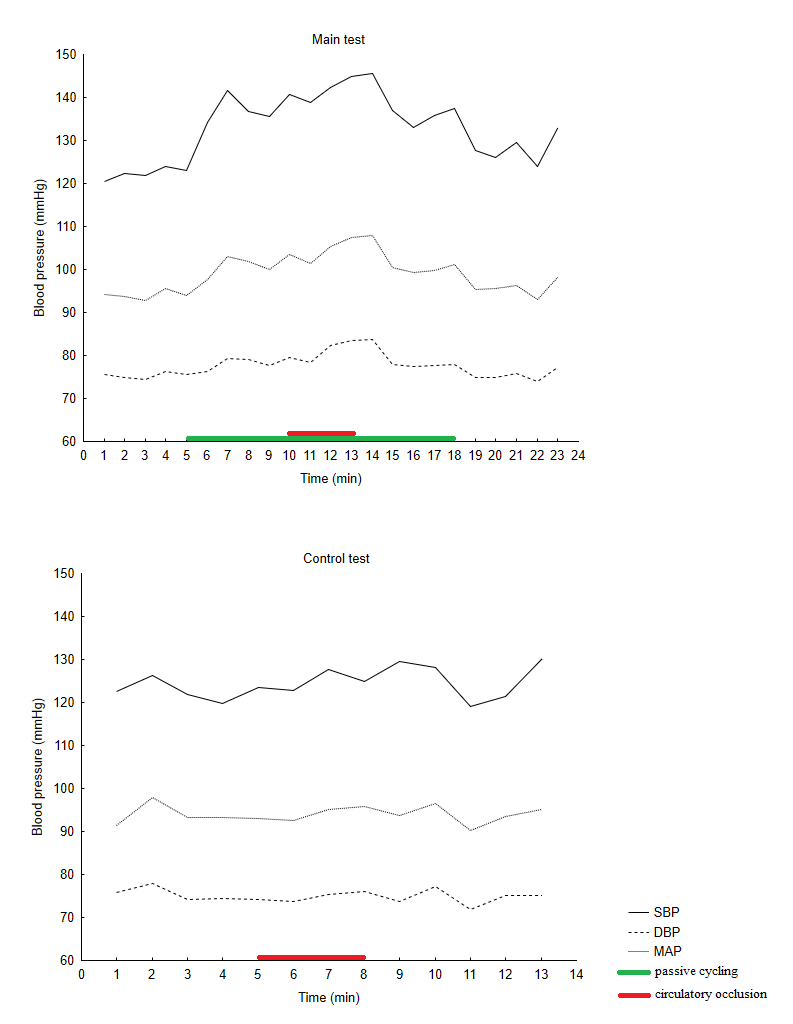


**Figure 2 Time course of total peripheral resistance**

**
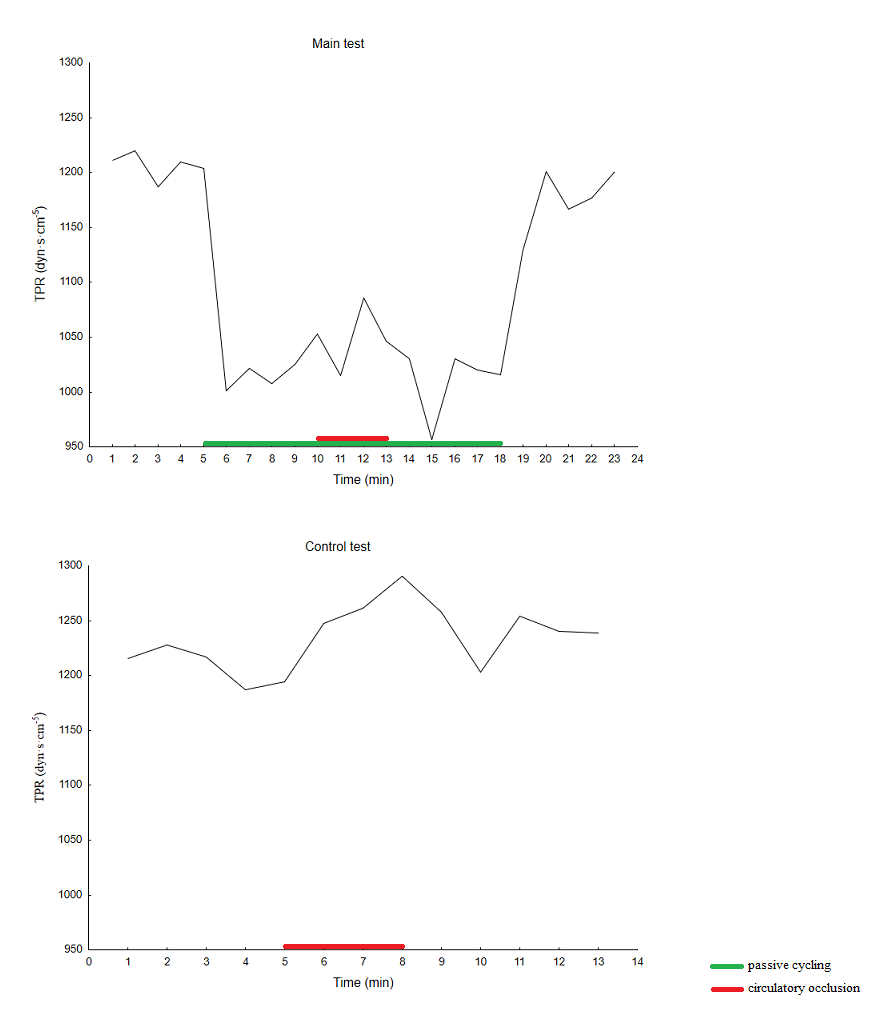
**

**Figure 3 Time course of stroke volume**

**
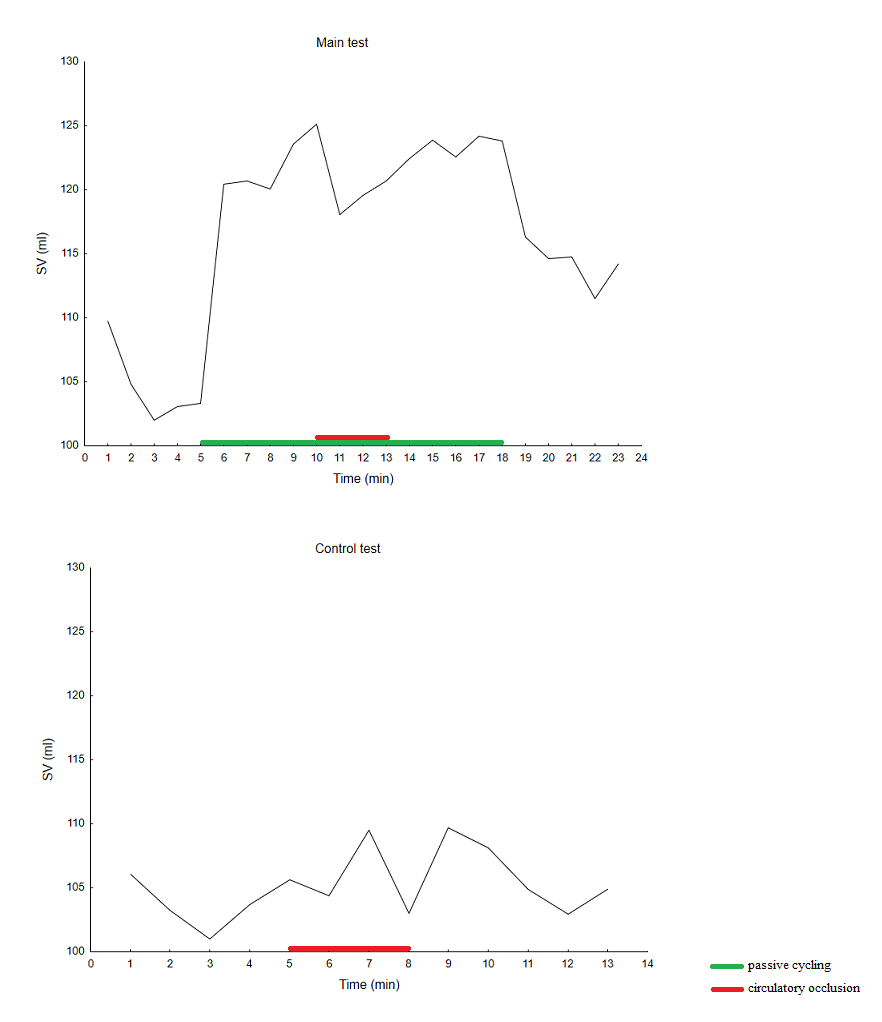
**

**Figure 4 Time course of heart rate**

**
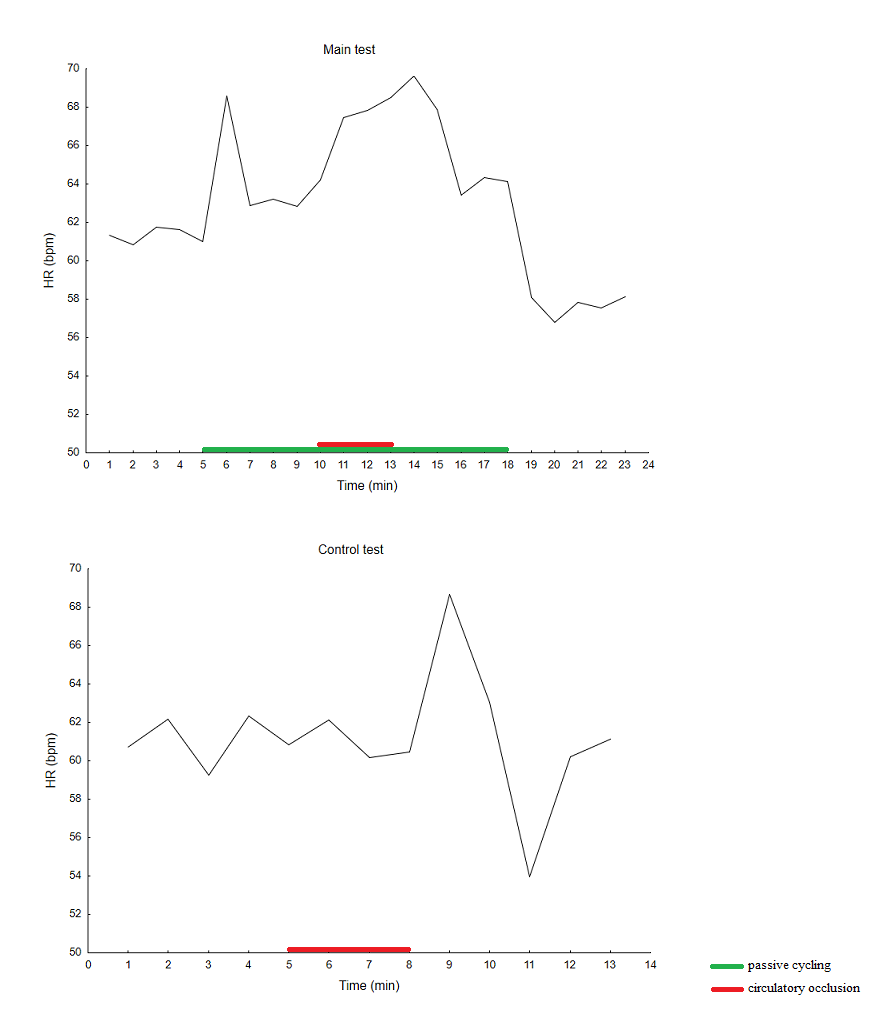
**

**Figure 5 Time course of tidal volume**

**
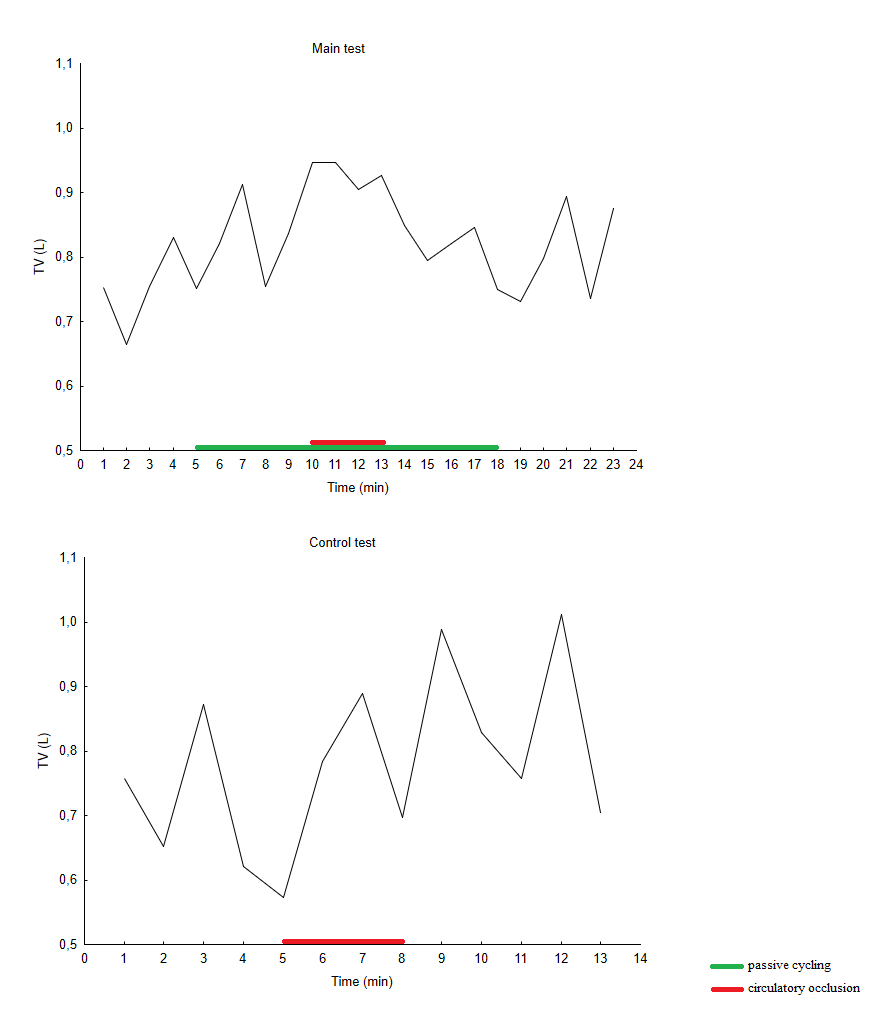
**

**Figure 6 Time course of breathing rate**

**
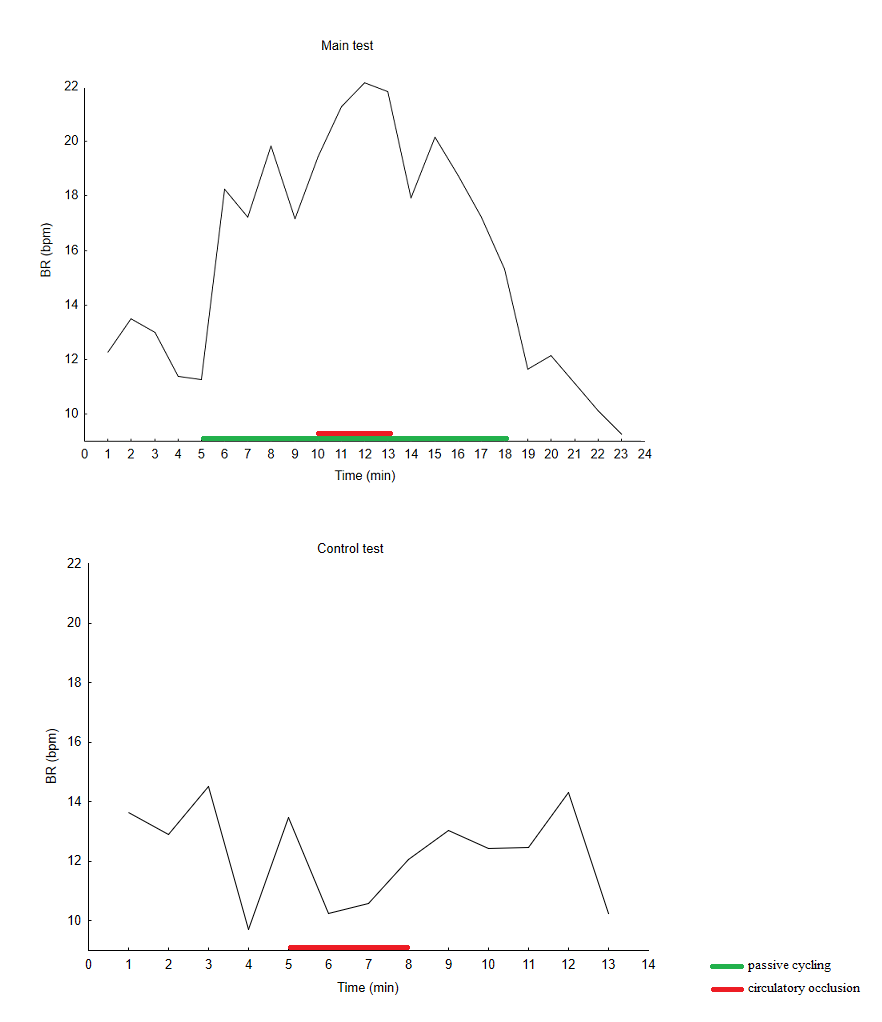
**

**Figure 7 Time course of minute ventilation**

**
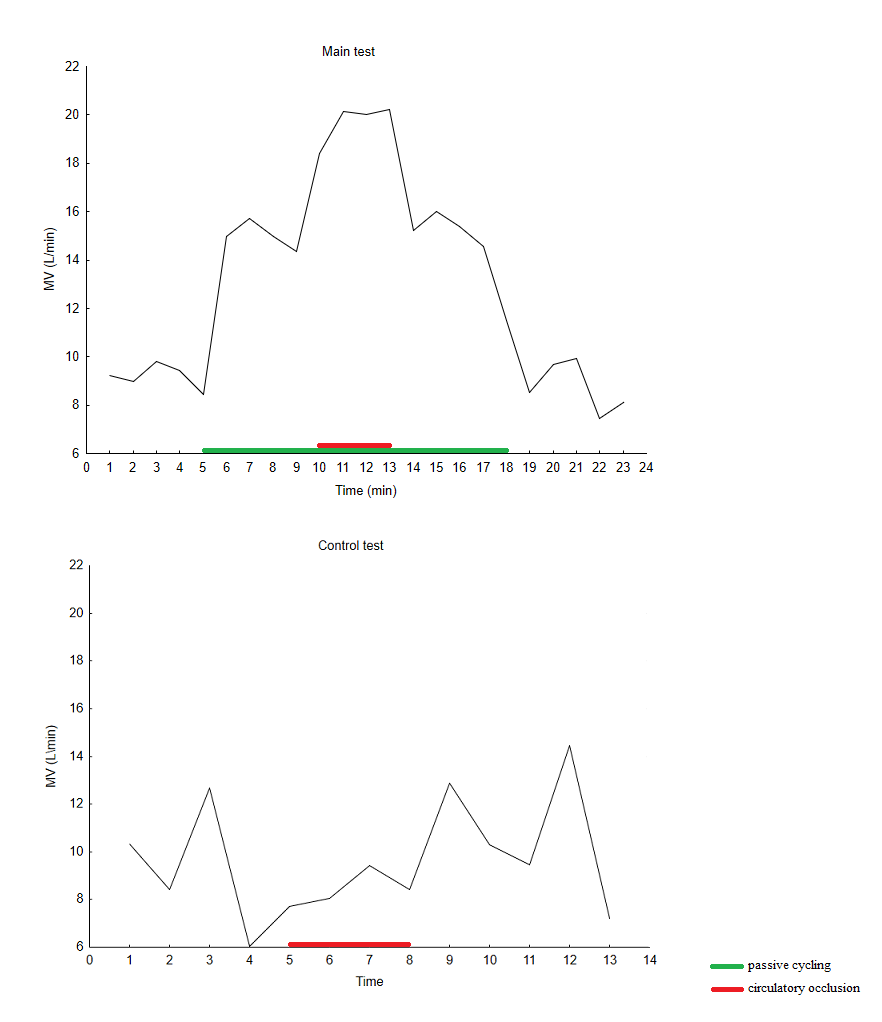
**
